# Association of single child family with subjective health complaints in children and adolescents

**DOI:** 10.1038/s41598-022-22618-x

**Published:** 2022-10-29

**Authors:** Hadith Rastad, Mostafa Qorbani, Kumars Pourrostami, Fatemeh Ochi, Ali Sheidayi, Hanieh-Sadat Ejtahed, Ehsan Seif, Nami Mohammadian Khonsari, Armita Mahdavi-Gorabi, Ramin Heshmat, Roya Kelishadi

**Affiliations:** 1 Cardiovascular Research Center, Alborz University of Medical Sciences, Karaj, Iran; 2 Non-Communicable Diseases Research Center, Alborz University of Medical Sciences, Karaj, Iran; 3 Department of Pediatrics, Alborz University of Medical Sciences, Karaj, Iran; 4 Student Research Committee, Alborz University of Medical Sciences, Karaj, Iran; 5 Department of Epidemiology and Biostatistics, School of Public Health, Tehran University of Medical Sciences, Tehran, Iran; 6 Obesity and Eating Habits Research Center, Endocrinology and Metabolism Clinical Sciences Institute, Tehran University of Medical Sciences, Tehran, Iran; 7 Chronic Diseases Research Center, Endocrinology and Metabolism Population Sciences Institute, Tehran University of Medical Sciences, Tehran, Iran; 8 Department of Pediatrics, Child Growth and Development Research Center, Research Institute for Primordial Prevention of Non-Communicable Disease, Isfahan University of Medical Sciences, Isfahan, Iran; 9 Social Determinants of Health Research Center, Alborz University of Medical Sciences, Karaj, Iran

**Keywords:** Psychology, Signs and symptoms

## Abstract

The number of single-child families has been increasing across developing countries during the last decades. We aimed to examine the association between being a single child (SC) and subjective health complaints (SHCs) in Iranian children and adolescents. This study was conducted as a part of the fifth survey of a national surveillance program entitled Childhood and Adolescence Surveillance and Prevention of Adult Non-communicable disease (CASPIAN-V). This national survey included a total of 14,400 students 7–18 years and their parents from rural and urban areas in 30 provinces of Iran. Data on demographic characteristics, lifestyle variables, and SHCs were measured using the questionnaire of the World Health Organization on Global School-based Health Survey (WHO-GSHS). Multivariate logistic regression models were used to estimate odds ratios (OR) and 95% confidence intervals (95%CI) for the association of being an SC with SHCs. Data on 14,151 participants were available for this study, of whom 7.7% (1092) were SCs. The most frequent SHCs were irritability (37.1%, 95%CI: 36.3–37.9%), feeling nervous 32.5%, 95% CI: (31.7–33.3%), and headache 24.3%, 95%CI: (23.6–25.0% ). In the multivariate model, being an SC significantly increased the odds of dizziness [adjusted OR (95% CI): 1.37(1.08–1.72)] and backache complaint [1.22(1.01–1.47)]. The association of being an SC with other SHCs (feeling low, irritability, feeling nervous, difficulty in getting to sleep, headache, stomachache) was not statistically significant (*p* value > 0.05). Our results suggest that being an SC may be associated with higher odds of dizziness and backache complaints.

Adolescents’ health status is affected by their behaviors and lifestyles, which also influence their later stages of life^1^; Nonetheless, surveillance systems have inadequately investigated this period, and there are limited data on the health status and health complaints of adolescents^2^. In this regard, subjective health complaints (SHCs), including psychological and somatic symptoms, have been reported to be prevalent among adolescents in developed countries^3,4^ and are also known to be a primary determinant of health services visits^5^. There is evidence that adverse health and psychosocial outcomes such as depression, anxiety, school absence, and bullying are more prevalent among adolescents with weekly health complaints^6–9^. Moreover, SHCs tend to persist into adulthood, resulting in chronic pain symptoms and psychological disorders later in life^10,11^.

Some family structure-related factors, such as birth order, are well-known to be associated with SHCs, but the role of a single-child (SC) family has hardly ever been investigated in this regard;^12^. However, as school-aged children in SC families are at an increased risk of mental health problems and obesity^12–15^, they might be at a higher risk of having SHCs.

During the last decades, only-child families becoming far more common in developing countries such as Iran, partly due to delayed parenthood among employed women and financial pressures, including childcare costs^16^. Since the association between the SC family and the SHCs is not well established yet, we conducted this study on school-aged children in Iran.

## Methods

This study was conducted according to the STROBE guideline^17^. We used the data from the fifth childhood and adolescence surveillance and prevention of adult non-communicable disease (CASPIAN-V) study, an ongoing national survey on school children in urban and rural areas of Iran^18^. The CASPIAN study started in 2003 and has been repeated every 2–3 years^19–22^. The design, methods of measurements, and sampling strategy of the CASPIAN-V survey have been described in detail elsewhere^18^. In brief, 14,400 school children and adolescents aged 7 to18 years were recruited from 30 provinces in Iran using a multistage, stratified, cluster sampling approach. Expert health care professionals conducted physical examinations^18^.

Demographic characteristics (such as age, sex, and the number of children in the family), lifestyle variables, and SHCs were collected using the Global School-based Health Survey (GSHS) questionnaire. The Persian version of the questionnaire mentioned above, with acceptable Cronbach’s alpha and Pearson’s correlation coefficients of 0.97 and 0.94, respectively, was used for this study^23^. Students were divided into two groups (“SC” or “with siblings”) based on the number of children in the family. All questionnaires were filled out confidentially under the supervision of trained nurses.

Students’ physical activity (PA) status was determined using data on their frequency of leisure-time PA outside the school during the week prior to data gathering using a validated questionnaire. Enough PA was considered at least 30 min exercise duration per day, leading to sweating and substantial increases in breathing or heart rate^24^. Each student’s screen time (ST) was measured using a questionnaire assessing the average number of hours/day spent watching TV/VCDs, personal computers, or electronic games (EG) separately on weekdays and weekends. The total cumulative spent time was summarized into main categories: “less than 2 h per day” (Low) and “2 h per day or more” (High)^25^.

The socioeconomic status (SES) of the students was assessed using data the following variables: parental education, parents’ job, possessing a private car, school type (public/private), and having a personal computer were combined using the principal component analysis (PCA) method as a unique index, then categorized into tertiles (low; intermediate and high SES)^26^.

SHCs were assessed using the validated GSHS questionnaire. Students were asked about the frequency of experiencing a variety of psychological (feeling low, irritable, nervous, and having difficulty in going to sleep) and somatic symptoms (headache, stomach ache, backache, feeling dizzy) during the past six months before the survey initiation. Response options for each item included: ‘about every day,’ ‘more than once a week,’ ‘about every week,’ ‘about every month’, and ‘rarely or never.’ The responses were categorized as “weekly or more” (Yes) and “rarely or never” (No)^27^.

### Statistical analysis

All statistical measures were estimated by survey data analysis methods using Statistical analysis Using the Stata package ver. 11.0 (Stata Statistical Software: Release 11. College Station, TX: Stata Corp LP. Package). The results are presented as mean ± standard deviation (SD), and frequency (percentage) for qualitative and quantitative variables, respectively. A student t-test was used to compare means, and the Chi-square test was used to determine the association between the categorical variables.

The association of SC status with SHCs was assessed using simple and multivariate logistic regression models. In the multivariate (adjusted) model, age, living area, sex, living with parents, duration of parent marriage, PA and ST, and SES were included as a confounder. The results of logistic regression models are presented as odds ratio (OR) and 95% CI. In all tests, a *p* value of < 0.05 was considered statistically significant.

### Ethics approval and consent to participate

The study protocol was reviewed and approved by the Research and Ethics Council of Isfahan University of Medical Sciences (Project number: 194049). We obtained signed written informed consent from schoolchildren under 16 years, and verbal informed consent from all parents/legal guardians and schoolchildren older 16 years

## Results

Out of 14,440 children and adolescents, the data of 14,151 participants (response rate: 98.3%) were available for the present study, of whom 7.7% (1092) were SCs.

Table 1 presents the overall and sex-stratified participants’ general characteristics and subjective health complaints. The mean age ± SD was 12.3 years ± 3.2, 50.7% (7172) were boys, and 71.4% lived in urban areas. Overall, the most prevalent SHCs were irritability (37.1%, 95%CI: 36.3–37.9%), feeling nervous (32.5%, 95%CI: 31.7–33.3%), headache (24.3%, 95% CI: 23.6–25.0%), and difficulty going to sleep (21.1%, 95% CI: 20.4–21.8%) respectively. A higher percentage of girls in comparison to boys reported having headaches (*p* value = 0.02), feeling dizzy (*p* value = 0.002), being irritable (*p* value = 0.02), and feeling nervous (*p* value = 0.001). Table 2 presents the participants' SHCs based on sex and sibling status. Overall, SCs, compared to those with siblings, significantly had a higher prevalence of dizziness (*P* value = 0.031) but a lower prevalence of feeling nervous (*p* value = 0.04). The prevalence of other subjective health complaints was not statistically different between the two groups. (all *p* values > 0.05). In stratification analysis by sex, none of the SHCs significantly differed across SCs and those with siblings, except for dizziness in females (*p* value = 0.007). In the univariate logistic regression model (Model I), being an SC significantly increased the odds of feeling dizzy [OR (95% CI): 1.26(1.02–1.57)]; but decreased the odds of feeling nervous [OR (95% CI): 0.87(0.76–0.99)]. In the multivariate model (adjusted model), being an SC significantly increased the odds of dizziness [OR (95% CI): 1.37 (1.08–1.72)] and backache complaints [OR (95% CI): 1.22 (1.01–1.47)]. (Table 3).

**Table 1:** General characteristics and subjective health complaints in participants: the CASPIAN-V study.

Variable	Total	Boy N = 7172	Girl N = 6979	*p* value
Mean age of students(year)^1^	12.3(3.2)	12.4 (3.1)	12.2 (3.2)	< 0.001*
Duration of parents’ marriage (year)	19.7(6.4)	19.8(6.3)	19.6(6.5)	0.13
**SC status**^**2**^				
SC	1092(7.7)	545(7.6)	547(7.8)	0.59
Children with siblings	13,059(92.3)	6627(92.4)	6432(92.2)	
**Living area**^**2**^				
Urban	10,106 (71.4)	5150(71.3)	5044(71.6)	0.66
Rural	4045 (28.6)	2078(28.7)	2002(28.4)	
Living with parents	13,329(94.1)	6751 (94.1)	6578 (94.1)	0.97
**Physical activity**^**2**^				
Low	8160 (58.2)	4020(56.2)	4215(60.4)	< 0.001*
High	5859 (41.8)	3138(43.8)	2768(39.6)	
**Screen time**^**2**^				
Low	13,067(92.5)	5863(83.4)	5781(84.3)	0.18
High	1065(7.5)	1164 (16.6)	1079(15.7)	
**SES**				
Low	4496 (33.2)	2325(33.6)	2234(33.3)	0.08
Medium	4510 (33.3)	2343(33.8)	2172(32.4)	
High	4544 (33.5)	2255(32.6)	2297(34.3)	
**SHCs**^**2**^				
Headache	3421 (24.3)	1689(23.4)	1758(25.0)	0.02*
Stomachache	2211 (15.7)	1103(15.3)	1126(16.1)	0.23
Backache	1907 (13.7)	938(13.2)	980(14.2)	0.11
Feeling dizzy	1107 (8.0)	518(7.3)	605(8.7)	0.002*
Irritability	5227 (37.1)	2604(36.5)	2671(38.4)	0.02*
Feeling low	1698 (12.2)	829(11.7)	881(12.7)	0.06
Difficulty in getting sleep	2936 (21.1)	1458(20.5)	1509(21.8)	0.06
Feeling nervous	4548 (32.5)	2224(31.2)	2358(33.9)	0.001*

SC: Single child, SES: Socioeconomic status, SHCs: Subjective health complaints.

^1^Data are presented as mean (standard deviation),

^2^Data are presented as numbers (percentage).

* Statistically significant.

**Table 2:** Frequency of subjective health complaints stratified by sex and sibling status: the CASPIAN-V study.

Variable	Total n(%)	SC N = 1092 n(%)	Having siblings N = 13,059 n(%)	*p* value
**Boy, N = 7172**				
Headache	1679 (23.5)	129 (23.7)	1550 (23.5)	0.910
Stomachache	1094 (15.3)	75 (13.8)	1019 (15.5)	0.315
Backache	931 (13.2)	76 (14.5)	855 (13.1)	0.382
Feeling dizzy	512 (7.3)	40 (7.5)	472 (7.2)	0.794
Irritability	2583 (36.5)	184 (34.8)	2399 (36.7)	0.390
Feeling low	823 (11.7)	60 (11.4)	763 (11.7)	0.840
Difficulty in getting sleep	1446 (20.5)	113 (21.4)	1333 (20.4)	0.594
Feeling nervous	2208 (31.2)	147 (27.6)	2061 (31.5)	0.065
**Girl, N = 6979**				
Headache	1742 (25.1)	138 (25.6)	1604 (25.0)	0.759
Stomachache	1117 (16.1)	88 (16.4)	1029 (16.1)	0.836
Backache	976 (14.2)	79 (15.1)	897 (14.2)	0.552
Feeling dizzy	595 (8.7)	62 (11.8)	533 (8.4)	0.007*
irritability	2644 (38.4)	198 (37.6)	2446 (38.5)	0.711
Felling low	875 (12.7)	57 (10.9)	818 (12.9)	0.190
Difficulty in getting sleep	1490 (21.7)	105 (20.0)	1385 (21.8)	0.340
Felling nervous	2340 (33.9)	167 (31.8)	2173 (34.1)	0.284
**Total, N = 14,151**				
Headache	3421 (24.3)	267 (24.6)	3154 (24.2)	0.762
Stomachache	2211 (15.7)	163 (15.1)	2048 (15.8)	0.579
backache	1907 (13.7)	155 (14.8)	1752 (13.6)	0.298
Feeling dizzy	1107 (8.0)	102 (9.7)	1005 (7.8)	0.031*
irritability	5227 (37.1)	382 (36.2)	4845 (37.5)	0.389
Felling low	1698 (12.2)	117 (11.2)	1581 (12.3)	0.281
Difficulty in getting sleep	2936 (21.1)	218 (20.7)	2718 (21.1)	0.764
Felling nervous	4548 (32.5)	314 (29.7)	4234 (32.8)	0.040*

SC: Single child.

* Statistically significant.

**Table 3:** Association between sibling status (SC vs. child with siblings) and subjective health complaints in logistic regression analysis: the CASPIAN-V study.

Variable	Model I OR (95% CI)	Model II OR (95% CI)
Headache	1.02 (0.88–1.18)	1.00 (0.86–1.18)
Stomachache	0.95 (0.80–1.13)	1.10 (0.91–1.34)
Backache	1.10 (0.92–1.31)	1.22 (1.01–1.47)*
Feeling dizzy	1.26 (1.02–1.57)*	1.37 (1.08–1.72)*
Irritability	0.94 (0.83–1.08)	0.97 (0.84–1.11)
Felling low	0.9 (0.73–1.09)	0.90 (0.72–1.11)
Difficulty in getting sleep	0.98 (0.84–1.14)	0.94 (0.79–1.10)
Felling nervous	0.87 (0.76–0.99)*	0.93 (0.80–1.09)

Model I: crude model, Model 2: adjusted for age, sex, living area, duration of parent marriage, living with a parent, SES, PA, and ST.

* Statistically significant.

## Discussion

Our findings also showed that SCs were more likely to complain of dizziness and backache than those with siblings after controlling for potential confounders. The prevalence of other SHCs, including headaches, stomachache, irritability, feeling low, and difficulty in going to sleep, were similar across these two groups.

In line with our study, Hesketh et al. noted that being an only child was not significantly associated with complaints of headache or abdominal pain^28^. Other studies have reported only a slight difference between SCs and those with siblings regarding psychological and health measures such as stress levels^29–32^. However, in another study conducted by Wang et al. on rural Chinese preschool children (3–6 years), SCs had a significantly higher score of somatic complaints compared to those with siblings; nonetheless, no significant differences were found between them regarding undesirable personality traits, including irritability, less independence, withdrawal, frustration proneness, and assertive behaviors^33^. Furthermore, in contrast to our findings, Yao et al. noted that SCs had a lower anxiety score, physical and mental suboptimal health , including perceived stress, sleep, pain, anxiety, and depression-symptoms, compared to children with siblings^34^. However, the differences across studied populations, such as nationality, age differences (pre-school/high school), and other residue factors, can be the cause of different findings across studies. The impacts of being an only child on psychosocial and somatic symptoms of children is controversial. Some studies indicate that anxiety, depression and the other aforementioned variables of SCs is lower compared to those with siblings; probably due to SCs receiving more attention from their parents, having more spiritual care, and being less of an economic burden ^34^; and stress the fact that the parents of these children devote more time, care and energy to them, thus providing a better guidance for their children;^35^, especially in some stressful situations^36^. Hence, these children’s psychosocial and somatic symptoms are more attended to than those with siblings. However, some studies, especially earlier studies on the subject, believed that the greater attention and higher devotion to single childes, alongside loneliness and other factors, result in significant stress and depression among SCs, resulting in various subjective health complaints^14,37^.

Moreover, our findings showed that approximately one of three to five school-aged children reported irritability (37.1%), feeling nervous, headache (24.3%), and/or difficulty going to sleep (21.1%). Some SHCs, including headaches, dizziness, irritability, and nervousness, were more frequent among girls than boys. This finding was in line with the findings of other studies which reported the high prevalence of SHCs among children and adolescents worldwide, with a wide variation among countries and a rising trend during the past decades^27,38–43^. Based on data from the 2017/2018 international Health Behaviour in School-aged Children survey (HBSC) among adolescents aged 11–15 years old (n = 228, 979) across 45 countries in Europe and North America, a significant number of participants, especially those living with low household SES reported SHCs symptoms, including a feeling irritable (~ 43%), feeling nervous (42%), sleep difficulties (35%), feeling low (30%), headache (29%), backaches (22%), abdominal pain (19%) and feeling dizzy (17%)^44^.

Our findings regarding the higher prevalence of SHCs among girls than boys were concordant with previous studies^27,44,45^. Aanesen et al. showed that lower self-esteem and stress among girls could justify this finding^45^. It has also been debated that females are more sensitive to their health and more willing to talk about their bodily experiences^27,46^.

Overall, the SHCs have the potential to be disabling and negatively influence school-aged children’s functional ability and school attendance^47^. furthermore, children with SHCs might have an increased risk of developing somatic and/or psychiatric illness in later life^43,47–49^.

## Limitations and strengths

This study had some limitations. The cross-sectional nature of the CASPIAN-V survey limited causality inference regarding the association between single-child status and childhood and adolescence SHCs. Hence to justify the findings of this study, longitudinal studies are needed. Another limitation is recall bias, in which subjects may not remember specific details such as the frequency of experiencing various psychological and somatic symptoms in the 6 months before the survey. Nevertheless, this study is the first Iranian study that uses a large national sample of children and adolescents across urban and rural areas to examine the association between being an SC and SHCs after controlling for potential confounders.

## Implications

The current study found some public health implications for children and adolescents SHCs. First, the frequency of SHCs among Iranian children and adolescents is relatively high, which suggests that urgent preventive or intervention strategies are needed. Secondly, higher prevalence of most of the SHCs among girls compared to boys indicates that special interventions are needed to reduce or prevent SHCs in this sex group. Lastly, the higher prevalence of some SHCs among SC families highlights the fact that in communities facing an increment of single-child families, some SHCs may increase. Therefore greater attention should be placed on psychosocial education and interventions around families with SC and gender equality.

## Generalizability

Our study was conducted on a large representative sample of Iranian children and adolescents. Therefore the result of this study can be generalized to all Iranian school-aged children.

## Conclusions

Our study shed some light on the association of SHCs with only-child families in a national sample of school-aged children in Iran. Our findings suggest that children and adolescents in an SC family might more frequently have a complaint of dizziness and backache. Nonetheless, more research, especially cohort studies, is needed to understand the underlying mechanisms between Sibling status and psychological status.

## Data Availability

The data used in the current study are available from the corresponding authors on reasonable request.

## Abbreviations

CASPIAN: Childhood and adolescence surveillance and prevention of adult non-communicable disease
CI: Confidence interval
EG: Electronic games
GSHS: Global School-based Health Survey
OR: Odds ratio
PA: Physical activity
PCA: Principal component analysis
SD: Standard deviation
SES: Socioeconomic status
ST: Screen time
SHCs: Subjective health complaints
SC: Single child
WHO-GSHS: World Health Organization on Global School-based Health Survey
